# Survival in Advanced-Stage Epithelial Ovarian Cancer Patients with Cardiophrenic Lymphadenopathy Who Underwent Cytoreductive Surgery: A Systematic Review and Meta-Analysis

**DOI:** 10.3390/cancers13195017

**Published:** 2021-10-07

**Authors:** Malika Kengsakul, Gatske M. Nieuwenhuyzen-de Boer, Anna H. J. Bijleveld, Suwasin Udomkarnjananun, Stephen J. Kerr, Christa D. Niehot, Heleen J. van Beekhuizen

**Affiliations:** 1 Department of Gynecologic Oncology, Erasmus MC Cancer Institute, University Medical Center Rotterdam, 3000 CA Rotterdam, The Netherlands; g.nieuwenhuyzen-deboer@erasmusmc.nl (G.M.N.-d.B.); h.vanbeekhuizen@erasmusmc.nl (H.J.v.B.); 2 Panyananthaphikkhu Chonprathan Medical Center, Department of Obstetrics and Gynecology, Srinakharinwirot University, Nonthaburi 11120, Thailand; 3 Department of Obstetrics and Gynecology, Albert Schweitzer Hospital, 3318 AT Dordrecht, The Netherlands; a.bijleveld@erasmusmc.nl; 4 Division of Nephrology, Department of Medicine, Faculty of Medicine, King Chulalongkorn Memorial Hospital, Chulalongkorn University, Bangkok 10330, Thailand; Suwasin.u@gmail.com; 5 Biostatistics Excellence Centre, Faculty of Medicine, Chulalongkorn University, Bangkok 10330, Thailand; Stephen.K@chula.ac.th; 6 Medical Library, Erasmus MC Cancer Institute, University Medical Center Rotterdam, 3000 CA Rotterdam, The Netherlands; c.niehot@erasmusmc.nl

**Keywords:** cardiophrenic lymph node, ovarian cancer, overall survival, progression-free survival

## Abstract

**Simple Summary:**

Favorable survival outcomes for patients with advanced-stage ovarian cancer are associated with complete cytoreduction. Enlarged cardiophrenic lymph node (CPLN) is commonly observed in advanced-stage epithelial ovarian cancer (AEOC); however, the prognostic impact of CPLN adenopathy is inconclusive. In this study, we evaluate the clinical outcomes of CPLN adenopathy in AEOC patients who underwent cytoreductive surgery. This systematic review and meta-analysis demonstrated that enlarged CPLN in preoperative imaging is highly associated with metastatic involvement. Patients with CPLN adenopathy had a significantly increased risk of recurrence of disease and dying from the disease in comparison to those without adenopathy, a finding likely related to more advanced disease in this group. Currently, there are no data that definitively demonstrate a therapeutic benefit of CPLN resection. Further randomized controlled trials should be conducted to definitively demonstrate whether CPLN resection at the time of cytoreductive surgery is beneficial.

**Abstract:**

Purpose: To evaluate the clinical outcomes of enlarged cardiophrenic lymph node (CPLN) in advanced-stage epithelial ovarian cancer (AEOC) patients who underwent cytoreductive surgery. Methods: The Embase, Medline, Web of Science, Cochrane Library, and Google Scholar databases were searched for articles from the database inception to June 2021. Meta-analysis was conducted to determine the prognostic impact of surgical outcome, postoperative complication, and survival using random-effects models. Results: A total of 15 studies involving 727 patients with CPLN adenopathy and 981 patients without CPLN adenopathy were included. The mean size of preoperative CPLN was 9.1± 3.75 mm. Overall, 82 percent of the resected CPLN were histologically confirmed pathologic nodes. Surgical outcomes and perioperative complications did not differ between both groups. The median OS time was 42.7 months (95% CI 10.8–74.6) vs. 47.3 months (95% CI 23.2–71.2), in patients with and without CPLN adenopathy, respectively. At 5 years, patients with CPLN adenopathy had a significantly increased risk of disease recurrence (HR 2.14, 95% CI 1.82–2.52, *p* < 0.001) and dying from the disease (HR 1.74, 95% CI 1.06–2.86, *p* = 0.029), compared with those without CPLN adenopathy. CPLN adenopathy was significantly associated with ascites (OR 3.30, 95% CI 1.90–5.72, *p* < 0.001), pleural metastasis (OR 2.58, 95% CI 1.37–4.82, *p* = 0.003), abdominal adenopathy (OR 2.30, 95% CI 1.53–3.46, *p* < 0.001) and extra-abdominal metastasis (OR 2.30, 95% CI 1.61–6.67, *p* = 0.001). Conclusions: Enlarged CPLN in preoperative imaging is highly associated with metastatic involvement. Patients with CPLN adenopathy had a lower survival rate, compared with patients without CPLN adenopathy. Further randomized controlled trials should be conducted to definitively demonstrate whether CPLN resection at the time of cytoreductive surgery is beneficial.

## 1. Introduction

Ovarian cancer is the second leading cause of death among gynecological malignancies [1]. According to the International Federation of Gynecology and Obstetrics (FIGO), the majority of women with epithelial ovarian cancer (EOC) patients are initially diagnosed with advanced stage III and IV disease [2,3]. The current standard management for patients with EOC includes surgical cytoreduction in combination with adjuvant platinum-based chemotherapy. An independent prognostic factor in EOC is the amount of residual disease following cytoreductive surgery [4,5]; the absence of residual disease is associated with the most favorable survival outcomes [5,6].

In 2014, FIGO published a new classification defining retroperitoneal lymph node metastasis between the inguinal ligament and the diaphragm as regional nodes, categorized as FIGO stage IIIA [7]. Over a decade, extensive pelvic and paraaortic systematic lymphadenectomy procedures have been performed with the intention of removing potentially metastatic retroperitoneal lymph nodes. However, the randomized lymphadenectomy in patients with advanced ovarian neoplasm (LION) trial showed no benefit of systematic lymph node dissection in patients with radiologically unsuspicious nodes [8]. 

Enlargement of cardiophrenic lymph nodes (CPLNs), also referred to paracardiac or supradiaphragmatic lymphadenopathy, is one of the most common manifestations of extra-abdominal disease in patients with FIGO stage IV [9]. CPLNs are located in fatty tissue at the basal portion of the mediastinum. Their afferent lymphatics arise from the pericardium, anterior thoracoabdominal wall, pleura, and diaphragm [9]. Current guidelines for ovarian cancer staging issued by the female imaging subcommittee of the European Society of Urogenital Radiology (ESUR) 2010 state that CPLN larger than 5 mm in short-axis diameter, seen on a computed tomography (CT) scan or magnetic resonance imaging (MRI) scan, should alert the clinician to metastatic involvement [10]. Although enlarged CPLNs are frequently observed during preoperative imaging, their prognostic impact is unclear. Holloway et al. demonstrated a significant association between peritoneal metastases and enlarged CPLN with a short-axis diameter >5 mm on CT scans. The presence of enlarged CPLN was found to be associated with poor survival, regardless of the presence of peritoneal metastases [9]. Currently, the surgical feasibility of CPLN resection has been reported [11,12]. However, there is still no consensus on the survival benefit of these issues. We, therefore, performed a systematic review and meta-analysis to summarize available evidence. The primary objective of the study was to evaluate the clinical outcomes of advanced-stage EOC patients with CPLN adenopathy vs. to those without adenopathy who underwent cytoreductive surgery. The secondary objective was to evaluate the potential role of CPLN resection in EOC patients who had also undergone cytoreduction.

## 2. Material and Methods

### 2.1. Data Sources and Searches

The methods in this review are described based on the Preferred Reporting Items for Systematic Reviews and Meta-Analyses (PRISMA) Checklist [13], the PRISMA-S extension to the PRISMA Statement for Reporting Literature Searches in Systematic Reviews [14], and meta-analysis of observational studies (MOOSE) checklist [15]. The study was registered in PROSPERO, Registration Number 276488. The search was developed in Embase.com and then translated to other databases. The search was carried out on 1 June 2021 in the databases Embase.com, Medline ALL via Ovid, Web of Science Core Collection, and the Cochrane Central Register of Controlled Trials via Wiley. Additionally, a search was performed in Google Scholar from which the 200 most relevant references were downloaded using the software Publish or Perish [16]. See Table S1 for more information. 

The search contained terms for (1) cardiophrenic lymph node and (2) ovarian cancer (Table S1). The searches in Embase, Medline, and Web of Science were limited to exclude conference papers and animal-only articles. No study registries were searched, but Cochrane Central retrieves the contents of ClinicalTrials.gov and the World Health Organization’s International Clinical Trials Registry Platform. No authors or subject experts were contacted, and we did not browse unindexed journals in the field. 

### 2.2. Study Selection

Studies published in the English language with adequate information according to our including criteria and the Strengthening the Reporting of Observational Studies in Epidemiology (STROBE) statement [17], were included in the review. The titles and abstracts retrieved from the search strategy were independently screened by two authors (M.K. and G.N.). Then, they retrieved and reviewed the full texts of the seemingly relevant articles. Any disagreements between M.K. and G.N. were resolved through discussion and arbitration by a third author (H.B.). The reference lists of retrieved articles were searched for possibly missed relevant studies. We included retrospective and prospective cohort studies as well as clinical trials that evaluated the survival of advanced-stage epithelial ovarian cancer patients, by preoperative CPLN adenopathy status, who underwent cytoreductive surgery for primary disease. Only studies that reported the associated factors and survival outcomes were included. Studies presenting results for patients treated only by surgery were excluded unless details regarding surgical outcomes—, e.g., residual disease or perioperative complications—were provided. Studies published as conference abstracts, narrative review, editorials, letters, and short communications were excluded

### 2.3. Data Extraction and Quality Assessment

Preoperative CPLN adenopathy was defined as CPLN ≥ 5 mm on the short axis, diagnosed by preoperative CT or MRI scan, or fluor-18-deoxyglucose (FDG) uptake in positron emission tomography/computer tomography (PET/CT) [10]. Study characteristics extracted were the following: name of the first author, year of publication, country, study center, sample size, and study design. Patient-related characteristics extracted were mean age, mean cancer antigen (CA)125, mean size of CPLN, percentage of histologically proven metastatic lymph nodes, FIGO stage, histology, American Society of Anesthesiologists (ASA) classification, surgical outcome, presence of extra-abdominal metastasis, and postoperative complication rate. Primary outcomes were median progression-free survival (PFS) time, median overall survival (OS) time, 5-year PFS, and OS probabilities. PFS was defined as the time elapsed between the date of diagnosis to the date of recurrence. Overall survival was defined as the time elapsed from diagnosis to the date of death or last follow-up. All survival outcomes and patient characteristics were extracted from patients with a primary diagnosis of advanced-stage ovarian cancer. Two authors (M.K. and G.N.) independently evaluated the quality and the risk of bias in the observational studies included in the meta-analysis based on The Newcastle-Ottawa Quality Assessment Scale [18], and discrepancies were resolved in the manner described for article review.

### 2.4. Data Synthesis and Analysis

Results were pooled using random-effects meta-analyses to compute weighted mean differences (WMD) of continuous variables and pooled odds ratios (ORs) of binary variables. The pooled estimations are displayed with 95% confidence intervals (CIs). If the mean and standard deviation (SD) were not provided in the original articles, the method described by Wan et al. was used for calculation [19]. Pooled median survival was analyzed by the method of Simes et al. [20]. To calculate the individual study hazard ratios (HR), we used the methods described by Tierney et al. [21]. Heterogeneity in study effect sizes was assessed using the *I^2^* index and Cochran’s Q-test. *I*^2^ indices of 25%, 50%, and 75% indicate mild, medium, and high heterogeneity, respectively. Categorical variables are presented as number and percentage and continuous variables were presented as mean ± SD. Each variable was considered statistical significance when a *p*-value < 0.05. Publication bias was formally assessed using the Egger test. The analyses were performed using Stata Statistical Software Release 16.1 (StataCorp LLC, College Station, TX, USA).

## 3. Results

### 3.1. General Characteristic

The initial search retrieved 1468 articles. After the removal of duplicates, the titles and abstracts of 957 articles were screened, and 45 full-text articles were retrieved for a comprehensive review. In total, 15 articles were included in the review based on inclusion and exclusion criteria (Figure S1). All studies had adequate quality for analysis according to The Newcastle-Ottawa Quality Assessment Scale (Table S2).

Thirteen articles described retrospective cohort studies [9,11,22,23,24,25,26,27,28,29,30,31,32,33], one was a case report [34], and one a case series [35]. The studies spanned the period from 1997 to 2020. CPLN adenopathy was defined as CPLN ≥ 5 mm in six studies, ≥7 mm in four studies, and >8 mm on preoperative scans and/or bulky lymph nodes during surgery in three studies, and >10 mm in a single study. One study did not mention a threshold cutoff to define CPLN adenopathy. From these studies, 727 patients with CPLN adenopathy and 981 patients without CPLN adenopathy were included in our analyses (Table S3). 

The pooled baseline characteristics of patients by the CPLN adenopathy group are presented in Table 1. The pooled mean age of patients with and those without CPLN adenopathy was 59.17 ± 11.87 years and 60 ± 12.64 years, respectively. There was no difference in pooled mean CA125 level between the two groups when results were available. Overall, 43 percent of each group had ASA classification ≥ 3. In each group, the majority of patients had high-grade serous carcinoma. In addition, 73% of patients with CPLN adenopathy had been diagnosed with FIGO stage IV, vs. 23% of patients without CPLN adenopathy. Note that stage IV disease was defined as metastases other than CLPN involvement, for example, liver or spleen parenchymal involvement, extra-abdominal metastases, etc. The proportion of ascites, pleural metastases, pleural effusion, upper abdominal metastases, pelvic and abdominal adenopathy, carcinomatosis peritonitis, and other extra-abdominal metastases were highest in the CPLN adenopathy group. The proportion of patients achieving complete abdominal cytoreduction did not differ between the groups. Surgical complexity—reflected by the description of procedures including diaphragmatic stripping, splenectomy, and hepatectomy—was higher in the CPLN adenopathy group. Despite this finding, only a slightly higher proportion of perioperative complications was found for the CPLN adenopathy group (26% vs. 23%). Eight studies provided survival outcomes [9,11,24,25,26,29,32,33]. The pooled median PFS time was 14.6 months (95% CI 4.9–24.4) vs. 27.8 months (95% CI 3.2–52.5) in patients with and without CPLN adenopathy, respectively. The pooled median OS time was 42.7 months (95% CI 10.8–74.6) vs. 47.3 months (95% CI 23.2–71.2), respectively.

### 3.2. Intervention

Regarding the impact of CPLN resection during cytoreductive surgery, nine studies reported the mean size of preoperative suspicious nodes [11,23,24,25,26,28,29,32,34]. The baseline characteristics are presented in Table 2. The mean size of preoperative CPLN was 9.1 ± 3.75 mm. Four studies evaluated the CPLN location; right, left, bilateral and midline, and unknown [25,28,31,34]. Nearly 50% of the enlarged nodes were located in the right mediastinum. In total, 194 patients with enlarged CPLN underwent CPLN resection. Among them, 82% of patients had histologically proven CPLN metastases (Table S4). The most commonly used surgical technique was the transdiaphragmatic approach, followed by VATS and the substernal approach, respectively. Four studies reported postoperative complications [24,27,28,35]. Overall, 30 percent of patients were diagnosed with Clavien–Dindo grade IIIa or above [36]. No studies reported 30-day mortality. Nine studies provided adequate data regarding survival outcomes [9,11,24,25,26,29,31,32,33]. The pooled median PFS time was 17.7 months (95% CI 7.9–27.4), and the median OS time was 54.7 months (95% CI 15.2–94.3) in patients who underwent CPLN resection, compared with 15.3 months (95% CI 3.5–27.1) and 35.6 months (95% CI 19.0–52.2), respectively, in patients with CPLN adenopathy but without CPLN resection.

### 3.3. Meta-Analysis

**Figure 1:**
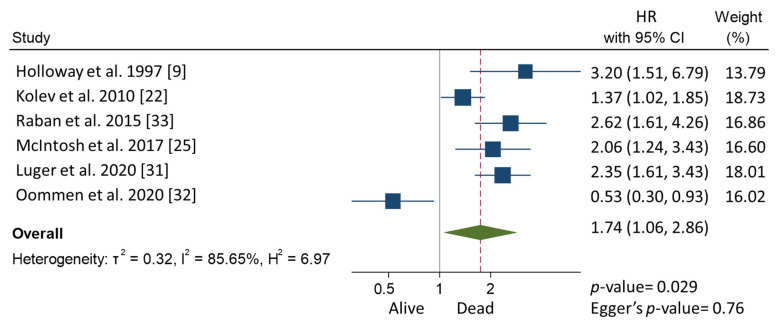
Forest plot displaying hazard ratios for mortality at 5 years in patients with CPLN adenopathy relative to those with no adenopathy.

**Figure 2:**
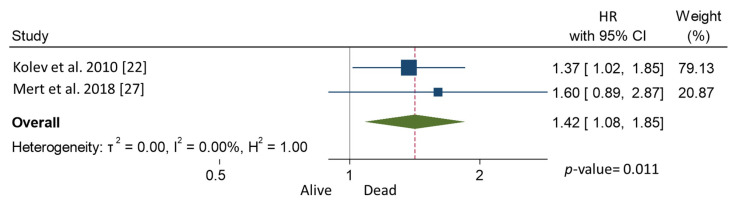
Forest plot displaying hazard ratios for mortality at 5 years after optimal abdominal cytoreduction surgery in patients with CPLN adenopathy relative to those with no adenopathy.

**Figure 3:**
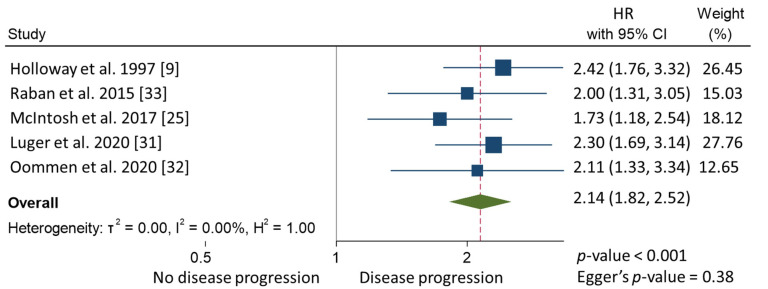
Forest plot displaying hazard ratios at 5-year disease-free survival in patients with CPLN adenopathy relative to those with no adenopathy.

In the meta-analysis, patients with CPLN adenopathy vs. patients without CPLN adenopathy had a significantly increased risk of ascites (OR 3.30, 95% CI 1.90–5.72), pleural metastases (OR 2.58, 95% CI 1.37–4.82), pleural effusion (OR 1.78, 95% CI 1.17–2.69), abdominal adenopathy (OR 2.30, 95% CI 1.53–3.46), and extra-abdominal metastases (OR 3.27, 95% CI 1.61–6.67). Neither the risk of upper abdominal metastases (OR 4.38, 95% CI 0.36–54.01) nor the prevalence of complete abdominal cytoreduction (OR 0.69, 95% CI 0.17–2.82) differed between groups (Table 3). Six studies provided 5-year overall survival rates [9,22,25,31,32,33] (Figure 1). Patients with CPLN adenopathy had a significantly increased risk of dying from the disease, compared with those without adenopathy (HR of death at 5 years; 1.74, 95% CI 1.06–2.86, *p* = 0.029). Two studies provided 5-year mortality in patients undergoing optimal abdominal cytoreductive surgery [22,27] (Figure 2). Those with CPLN adenopathy had a 1.42 times higher risk of dying from the disease than those without adenopathy (95% CI 1.08–1.85, *p* = 0.011). The forest plot displaying hazard ratios at 5-year disease-free survival in patients with CPLN adenopathy relative to those with no adenopathy is displayed in Figure 3. Funnel plots of these results are presented in Supplementary Data (Figures S2–S4). Unfortunately, data to inform survival times and probabilities in patients undergoing abdominally complete cytoreductive surgery in relation to CPLN resection were not provided in the individual studies. 

## 4. Discussion

Among advanced-stage EOC, 12–33% of patients will present with extra-abdominal and or visceral metastases [37]. Evidence generated over decades of research shows that complete cytoreduction at primary surgery is the cornerstone of favorable survival outcomes for advanced-stage ovarian carcinoma [6,38,39]. Of 208 patients with stage IV EOC from seven referral centers in France, patients on whom no operation was performed had significantly worse overall survival than those who had undergone primary debulking surgery or interval debulking surgery after neoadjuvant chemotherapy (NACT). The authors concluded that the presence of distant metastases should not deter surgeons from performing a radical procedure if it could be tolerated by the patient. In this study, patients who received chemotherapy only were in poor general condition and judged not suitable for surgery, or developed progressive disease during chemotherapy [40].

In the studies included in this review, the prevalence of radiologically suspected CPLN adenopathy varied from 11 to 50% in advanced-stage EOC. Three studies described suspected CPLN when the size was ≥5 mm in short-axis diameter according to ESUR2010 [22,27,31]. Luger et al. demonstrated that CPLN ≥ 5 mm in short axis correctly predicted carcinomatosis of the upper abdomen in 94% of cases [31]. In our meta-analysis, we additionally found that patients with CPLN adenopathy had a significantly increased likelihood of having ascites, abdominal adenopathy, and extra-abdominal metastases, compared with patients without CPLN adenopathy. We found that 82.4% of patients who underwent CPLN resection had pathologically confirmed metastatic lymph nodes. It must be noted that cutoff sizes for defining enlarged CPLN, and different diagnostic imaging modalities were included in our analysis. The percentage of pathologic lymph nodes found on imaging varied from 57.1% to 94.4%. Kim et al. purposed that the threshold of 7 mm in a short axis on CT scans was the optimal cutoff for predicting CPLN metastasis, with 63% sensitivity and 83% specificity [23]. Another study also reported the probability of detecting CPLN metastases of approximately 85% if the short axis of the node exceeded 7 mm [33]. In contrast, Pinelli et al. found metastases in only 57.14% of CPLN after interval debulking surgery (IDS) when using the same cutoff value. However, all patients in the study had received 3–6 cycles of NACT before IDS. The authors suggested that the lower detection rate was a consequence of chemotherapy-induced fibrosis after tumor regression [31]. Surprisingly, Cowan et al. reported the highest detection rate when the cutoff size was >5 mm in the short axis [24]. However, the mean size of CPLN in this study was as large as 15 ± 0.5 mm, while in other studies, the mean size ranged from 7 to 10 mm [23,26,29]. Lopes et al. reported that 85.7% of EOC patients were upstaged from stage IIIC to IV, based on histologically confirmed CPLN metastases when CPLN were ≥8 mm on preoperative imaging and/or exhibited 18-fluorine fluorodeoxyglucose uptake on PET/CT. Interestingly, all patients with PET/CT uptake in CPLN in this study— including those who had received NACT—had histologically positive lymph nodes [11]. Currently, many studies have demonstrated that PET/CT imaging increases the likelihood of finding CPLN metastases, in comparison with CT imaging, especially when they are <10 mm in size [26,27,41]. However, another study suggested that CPLN size ≥ 7 mm in a short axis on CT imaging has 85.7% positive predictive value and 58.8% negative predictive value [23], which is useful in settings where PET/CT is not always available. It also implies that PET/CT may be best reserved for patients with an unclear diagnosis, or for patients who have already received chemotherapy.

In some centers, gynecologic oncologists employ CPLN resection—most commonly with a transdiaphragmatic approach—with the aim to maximize the degree of cytoreduction. Many advantages of transdiaphragmatic approaches have been described. First, the procedure is feasible for both gynecologic oncologists and surgeons. Second, the patient’s position during surgery need not be changed. Third, it is not always necessary to insert a chest tube after the procedure [42,43]. In 2017, Cowan et al. reported on the largest cohort of EOC patients who underwent CPLN resection at primary cytoreductive surgery [24]. This cohort included patients who underwent CPLN resection via VATS and a transdiaphragmatic approach by a gynecologic oncologist or another consulting surgeon. The postoperative complication rate was 35%. Only 7% of the major postoperative complications were of a pulmonary nature—namely, pulmonary embolism, chylothorax, pleural effusion, and acute respiratory disease syndrome [24]. In our analysis, about 30% of CPLN-resected patients had ≥Clavien–Dindo grade IIIa. The authors of the various publications concluded, however, that the procedure was feasible, the morbidity rate acceptable, and no procedure-related deaths had occurred [23,24,28,34,44].

Although CPLN adenopathy is associated with extensive intra- and extra-abdominal tumor burden, it does not affect the surgical outcome. In our pooled analysis, abdominally complete cytoreductive surgery was accomplished in nearly 60% of patients with or without CPLN adenopathy. Notably, all studies had been conducted in tertiary centers, and surgery was highly complex in 65–85% of the patients. Our findings confirm that CPLN adenopathy is associated with a lower PFS and worse survival prognosis. On the other hand, the pooled median PFS and the OS of patients who underwent CPLN resection was superior to those of patients with CPLN adenopathy but without CPLN resection, suggesting a possible benefit of CPLN resection. Unfortunately, only two studies had directly compared survival outcomes in patients by whether or not they had CPLN resection. The authors reported no significant benefit of CPLN resection with regard to PFS and OS [26,29]. 

### Strength and Limitations

To our knowledge, this is the first systematic review and meta-analysis describing the clinical impact of CPLN adenopathy in patients with ovarian cancer. Still, some limitations of the study need to be addressed. First, most of the included studies were retrospective cohort studies, which carry the risk of missing data, and the possibility that significant biases may have occurred in the selection of controls. Second, although we presented the pooled median survival times in our study, these values could not be derived from every patient in all studies in the meta-analysis, because of inadequate information reported in the original studies. Third, adequate data regarding the overall survival of patients who underwent abdominally complete cytoreductive surgery and CPLN resection were not yet available for meta-analysis.

## 5. Conclusions

This systematic review and meta-analysis demonstrated that patients with CPLN adenopathy had a significantly higher risk of having ascites and intra- and extra-abdominal metastases than patients without CPLN adenopathy. Furthermore, PET/CT increases the detection rate of pathological CPLN metastases. CPLN exceeding 7 mm in the short axis on CT or MRI scans, with a positive predictive value for detecting metastatic lesions of approximately 85%. Based on these lines of evidence, it would seem appropriate that patients with CPLN of this size should therefore be considered candidates in future studies of the prognostic importance of CPLN dissection. PET/CT imaging should be reserved for patients with an inconclusive diagnosis, or a specific group of patients, for example, those who have received chemotherapy before imaging or patients in a healthcare setting where resources are limited. Patients with CPLN adenopathy had a significantly increased risk of recurrence of disease and dying from the disease in comparison to those without adenopathy. Currently, there are no data that definitively demonstrate a therapeutic benefit of CPLN resection, although this review and meta-analysis indicated a possible benefit of CPLN resection. For now, we recommend that EOC patients with suspected CPLN involvement be referred to a gynecologic oncologist to receive maximal cytoreduction. A randomized controlled trial should be conducted to demonstrate the potential benefit of CPLN debulking in cytoreductive surgery.

## Figures and Tables

**Table 1:** Pooled baseline characteristics of patients included in the systematic review.

Variable	CPLN Adenopathy	Number of Studies Total Study *n* = 15	Available Data of Patients *n* = 727	No-CPLN Adenopathy	Number of Studies Total Study = 9	Available Data of Patients *n* = 981
General						
Mean age, years ± SD	59.17 ± 11.87	10	350	60 ± 12.64	6	678
Mean CA125, U/dL ± SD	1166.36 ± 1259.22	8	278	1140.86 ± 1423.16	4	190
Anesthesiologists’ score ≥ 3	49 (43.36)	4	113	166 (43.57)	2	381
FIGO Stage IV * (other than CPLN)	145 (73.23)	6	198	164 (23.26)	7	705
High grade serous carcinoma	364 (85.85)	8	424	657 (81.92)	7	802
Complete abdominal cytoreduction	248 (59.05)	7	420	421 (59.21)	5	711
Optimal abdominal cytoreduction	169 (50.30)	7	336	275 (39.85)	6	690
High surgical complexity **	92 (85.98)	4	107	250 (65.62)	7	381
Perioperative complication ≥ Clavien-Dindo grade IIIa	36 (26.08)	6	138	86 (22.57)	2	381
Metastatic disease						
Ascites	441 (92.45)	8	477	284 (80.45)	4	353
Pleural metastases	40 (27.97)	3	143	21 (11.73)	2	179
Pleural effusion	104 (25.81)	5	403	50 (19.76)	2	253
Upper abdominal metastases	250 (65.27)	4	383	82 (26.28)	3	312
Pelvic adenopathy	67 (19.14)	5	350	36 (12.24)	3	294
Abdominal adenopathy	106 (27.04)	6	392	46 (14.74)	3	312
Carcinomatosis peritonii	315 (73.94)	6	426	179 (60.88)	3	294
Other extra abdominal metastases	46 (13.06)	4	352	20 (11.17)	2	179
Survival outcome						
Median OS, months (95%CI)	42.7 (10.8–74.6)	6	188	47.3 (23.2–71.3)	4	258
Median PFS, months (95%CI)	14.6 (4.9–24.4)	8	327	27.8 (3.2–52.4)	7	549

CPLN, cardiophrenic lymph nodes; * patient with CPLN adenopathy, stage IV disease was due to combination of other metastases, e.g., parenchymal involvement of liver or spleen, extra-abdominal metastases, etc.; ** high surgical complexity, e.g., diaphragmatic stripping, splenectomy, hepatectomy, etc. Continuous variables are shown as mean ± standard deviation, and categorical variables as frequency (and percentage).

**Table 2:** Pooled baseline characteristic of patients who underwent CPLN resection.

Variable	Value	Number of Studies Total = 9	Number of Patients Total = 2.09
General			
Mean size of CPLN *, mm ± SD	9.16 ± 3.75	9	471
Location *, total 152			
Right	73 (48.03)	4	152
Left	21 (13.82)		
Bilateral	20 (13.16)		
Midline	22 (14.47)		
Missing	16 (10.53)		
Surgical techniques, total = 100			
Video-assisted thoracic surgery	6 (6)	5	100
Transdiaphragmatic approach	90 (90)		
Substernal approach	4 (4)		
Pathologic CPLN node	160 (82.47)	7	194
Perioperative complication ≥ Clavien-Dindo grade IIIa	30 (30.30)	4	99
Survival outcome			
Median OS, months (95%CI)	54.7 (15.2–94.3)	3	93
Median PFS, months (95%CI)	17.7 (7.9–27.4)	4	145

CPLN, cardiophrenic lymph nodes; * CPLN adenopathy with and without resection. Continuous variables are shown as mean ± standard deviation, and categorical variables as frequency (and percentage).

**Table 3:** Meta-analysis of clinical characteristics of patients with CPLN adenopathy, compared with patients without CPLN adenopathy.

Variables	Number of Studies	Number of Patients	Odds Ratio with 95% CI	*p*-Value	I^2^ Index %	Q-Test *p*-Value = 0.567	Egger’s Test *p*-Value
Metastatic disease							
Ascites	4	735	3.30 (1.90–5.72)	<0.01	0	0.771	0.709
Pleuaral metastases	2	313	2.58 (1.37–4.82)	0.003	0	0.441	–
Pleural effusion	2	562	1.78 (1.17–2.69)	0.007	0	0.642	–
Upper abdominal metastases	3	663	4.38 (0.36–54.01)	0.249	97	<0.001	0.612
Pelvic adenopathy	3	634	1.73 (0.82–3.64)	0.145	59	0.089	0.320
Abdomimal adenopathy	3	663	2.30 (1.53–3.46)	<0.001	0	0.916	0.683
Carcinomatosis peritonii	3	634	2.02 (0.38–10.66)	0.406	90	<0.001	0.480
Extra abdominal metastases	2	313	3.27 (1.61–6.67)	0.001	0	0.975	–
Surgical outcome							
Completely abdominally cytoreduction	4	961	0.69 (0.17–2.82)	0.604	92	<0.001	0.085
Optimally adbominallly cytoreduction	5	924	1.32 (0.55–3.20)	0.532	80	<0.001	0.451

CPLN; cardiophrenic lymph nodes.

## Supplementary Materials

The following are available online at https://www.mdpi.com/article/10.3390/cancers13195017/s1, Table S1: The search strings, Table S2: Quality assessment of the included studies using the Newcastle-Ottawa Quality Assessment Scale, Table S3: Review of literatures, Table S4: The association between radiologically suspicious CPLN and histologically confirmed CPLN, Figure S1: Flow diagram of study selection, Figure S2: Funnel plot, using data from 6 studies of 5-year mortality in advanced stage EOC with CPLN adenopathy, Figure S3: Funnel plot, using data from 2 studies of 5-year mortality in advanced stage EOC with CPLN adenopathy after optimal abdominal cytoreductive surgery, Figure S4: Funnel plot, using data from 5 studies of 5-year disease free survival in advanced stage EOC with CPLN adenopathy.

## Abbreviations

ASA	American Society of Anesthesiologists
AEOC	Advanced-stage epithelial ovarian cancer
CPLN	Cardiophrenic lymph nodes
CT	Computed tomography scan
EOC	Epithelial ovarian cancer
ESGO	European Society of Gynecological Oncology
ESUR	European Society of Urogenital Radiology
FIGO	International Federation of Gynecology and Obstetrics
IDS	Interval debulking surgery
NACT	Neoadjuvant chemotherapy
MRI	Magnetic resonance imaging scan
OS	Overall survival
PFS	Progression-free survival
VATS	Video-assisted thoracic surgery
