# Supplementary material for: Survival in Advanced-Stage Epithelial Ovarian Cancer Patients with Cardiophrenic Lymphadenopathy Who Underwent Cytoreductive Surgery: A Systematic Review and Meta-Analysis
Source: Cancers (Basel). 2021 Oct 7;13(19):5017. doi: 10.3390/cancers13195017 (PMC8507882; doi:10.3390/cancers13195017)
Supplement: Supplementary file 1 [file cancers-13-05017-s001.zip › cancers-1381067-Supplementary material for XML update.pdf]

Supplementary Materials: Survival in Advanced-Stage Epithelial Ovarian Cancer Patients with Cardiophrenic Lymphadenopathy Who Underwent Cytoreductive Surgery: A Systematic Review and Meta-Analysis

Malika Kengsakul, Gatske M. Nieuwenhuyzen-de Boer, Anna H. J. Bijleveld, Suwasin Udomkarnjananun, Stephen J. Kerr, Christa D. Niehot and Heleen J. van Beekhuizen

Table S1. The search strings.

Source	Via	Years of coverage	Search string	Records
Embase	Embase.com	1971 - Present	('lymphadenopathy'/de OR 'cardiophrenic lymph node'/de OR (lymphadenopath* OR adenopath* OR ((lymph*) NEAR/3 (node*) NEAR/3 (enlarg* OR swollen OR swel*)) OR ((cardiophrenic* OR precardiac* OR pre-cardiac*) NEAR/3 (lymph*))):ab,ti,kw) AND ('ovary cancer'/exp OR 'ovary tumor'/de OR (((ovar*) NEAR/3 (cancer* OR tumor* OR tumour* OR neoplas* OR carcin*))):ab,ti,kw) NOT ([Conference Abstract]/lim) NOT ((animal/exp OR animal*:de OR nonhuman/de) NOT ('human'/exp))	703
Medline ALL	Ovid	1946 - Present	("Lymphadenopathy"/ OR (lymphadenopath* OR adenopath* OR ((lymph*) ADJ3 (node*) ADJ3 (enlarg* OR swollen OR swel*)) OR ((cardiophrenic* OR precardiac* OR pre-cardiac*) ADJ3 (lymph*))).ab,ti,kw.) AND (exp "Ovarian Neoplasms"/ OR (((ovar*) ADJ3 (cancer* OR tumor* OR tumour* OR neoplas* OR carcin*))).ab,ti,kw.) NOT (news OR congres* OR abstract* OR book* OR chapter* OR dissertation abstract*).pt. NOT (exp animals/ NOT humans/)	58
Web of Science Core Collection*	Web of Knowledge	1975 - Present	TS=(((lymphadenopath* OR adenopath* OR ((lymph*) NEAR/2 (node*) NEAR/2 (enlarg* OR swollen OR swel*)) OR ((cardiophrenic* OR precardiac* OR pre-cardiac*) NEAR/2 (lymph*)))) AND ((((ovar*) NEAR/2 (cancer* OR tumor* OR tumour* OR neoplas* OR carcin*)))) NOT ((animal* OR rat OR rats OR mouse OR mice OR murine OR dog OR dogs OR canine OR cat OR cats OR feline OR rabbit OR cow OR cows OR bovine OR rodent* OR sheep OR ovine OR pig OR swine OR porcine OR veterinar* OR chick* OR zebrafish* OR baboon* OR nonhuman* OR primate* OR cattle* OR goose OR geese OR duck OR macaque* OR avian* OR bird* OR fish*) NOT (human* OR patient* OR women OR woman OR men OR man))) AND DT=(Article OR Review OR Letter OR Early Access)	41
Cochrane Central Register of Controlled Trials	Wiley	1992 - Present	((lymphadenopath* OR adenopath* OR ((lymph*) NEAR/3 (node*) NEAR/3 (enlarg* OR swollen OR swel*)) OR ((cardiophrenic* OR precardiac* OR pre-cardiac*) NEAR/3 (lymph*))):ab,ti,kw) AND ((((ovar*) NEAR/3 (cancer* OR tumor* OR tumour* OR neoplas* OR carcin*))):ab,ti,kw)	3
Google Scholar			'pre cardiac lymph'|lymphadenopathy|'cardiophrenic lymph' "ovarian cancer|tumor|tumour|carcinoma|neoplasm swollen|enlarge|enlargement	152

Table S2. Quality assessment of the included studies using the Newcastle-Ottawa Quality Assessment Scale.

Authors	Selection (From total of ●●●●)	Comparability (From total of ●●)	Outcome (From total of ●●●)
Holloway J. B. et al. 1997	●●●●	●●	●●●
Kolev V. et al. 2010	●●●●	●●	●●●
Raban O. et al. 2015	●●●●	●●	●●
Kim H. T. et al. 2016	●●●●	●●	●●
Cowan R. A. et al. 2017	●●●●	●●	●●●
McIntosh J. L. et al. 2017	●●●●	●●	●●●
Luger K. A. et al. 2020	●●●●	●●	●●●

Mert I. et al. 2018	●●●●	●●	●●
LeeE O. I. et al. 2018	●●●●	●●	●●
Nuruzzaman Md. S. H et al. 2018	●●●●	●●	●●
Salehi S. et al. 2018	●●●●	●●	●●
Lopes A. et al. 2019	●●●●	●●	●●●
Prader S. et al. 2019	●●●●	●●	●●●
Pinelli C. et al. 2019	●●●●	●●	●●
Oommen I. et al.2020	●●●●	●●	●●●

Table S3. Review of literatures.

Reference No.	Studies	Study design	Patients (Enlarged CPLN)	FIGO	Study center	Definition of CPLN adenopathy	Objective	Results
9	Holloway J. B. et al. 1997	Retrospective cohort	78 (9)	II, III, recurrent stage I	Royal Marsden NHS, UK	> 5 mm. in short axis diameter on CT scan.	To identify progression free survival (PFS) and overall survival	The presence of CPLN enlargement is a significant adverse prognostic factor for both PFS and OS. The diagnosis of involved CPLN is important as it can define patients having stage IV as opposed to stage III disease.
22	Kolev V. et al. 2010	Retrospective cohort	212 (92)	IIIA-IV	Memorial Sloan Kettering Cancer Center	>5 mm. on the largest perpendicular measurements on CT scan	To determine prognostic significance of enlarge supradiaphragmatic nodes (SdLNs) noted on preoperative CT scan	All patients underwent PDR without SdLNs. 92 patients had SdLNs adenopathy. Median survival was 50 months for patients without adenopathy and 45 months for patients with adenopathy (P=0.09). In optimal cytoreduction group, median survival was 55 months for patients without adenopathy and 50 months for patient with SdLNs adenopathy. 73% were optimally debulked. There was no difference in the nodal size distribution based on cytoreductive status (P=0.36).
33	Raban O. et al. 2015	Retrospective cohort	72 (31)	III	Rabin medical Center, Israel	>10 mm. in short axis diameter on CT scan	To examine progression free interval and overall survival	No difference between groups in optimal cytoreduction rate. The median PFS for patients with CPLN was 9.0 vs 24 months (p =0.0097) and overall survival OS was 31.7 vs 61.3 months (p =0.001).
23	Kim H. T. et al. 2016	Retrospective cohort	31 (31)	III-IV	Seoul National University Hospital, Korea	>7mm. in short axis diameter on CT scan	To determine whether the preoperative diameter of CPLN the short and long axes, determined via CT scan, predicts pathological proved metastasis.	All patients with primary advanced ovarian cancer had CPLN dissection. The probability of detecting CPLN metastasis was approximately 85% when the short axis of the CPLN was > 7mm in preoperative CT scans.
24	Cowan R. A. et al. 2017	Retrospective cohort	54 (54)	IIIB-IV	Memorial Sloan Kettering Cancer Center, USA	>5 mm. in short axis diameter on CT scan	To examining its feasibility, safety, and potential impact on clinical outcome	All patients underwent CPLN resection. 94% had CPLN positive for metastasis. 56%were complete cytoreduction. Median PFS was 17.2 months (95%CI 12.6-21.8) and median OS was 70.1 months. (95%CI 51.2-89.0).
25	McIntosh J. L. et al. 2017	Retrospective cohort	88 (38)	III	Brigham and women's hospital, USA	≥ 5 mm. in short axis diameter on CT scan	to identify the optimal size threshold and to assess the prognostic significance of CPLN.	5mm. short-axis size threshold was associated with significantly decreased PFS (median 14 months, IQR11-17 vs 23 months, IQR12-59; P=0.02) and OS (median 44 months, IQR 27-69 vs 65 months, IQR 45-96; p=0.003). On Cox proportion hazards analysis, CPLN lymphadenopathy was significantly associated with decreased PFS (p=0.02) and OS (P=0.008).
26	Lee O. I. et al. 2018	Retrospective cohort	176 (50)	IIIB-IVB	Yonsei university college of	F-FDG uptake	To determine prognostic significance of enlarge	All patients underwent primary debulking surgeries. PFS (p=0.671) and OS (P=0.525) did not differ significantly between patients with PET/CT IV with SdLNM and PET/CT IV other

					medicine, South Korea		supradiaphragmatic nodes detected (SdLNM) by PET/CT.	metastases. However, patients with PET/CT IV with SdLNM had significantly decreased PFS (14 months, p<0.001) and OS (31.5 months=0.016) than those with PET/CT stageIII (PFS 18 months, median OS 37.5 months). Debulking lesions did not improve PFS (p=0.425) and OS (p=0.465) of patients with AEOC.
27	Mert I. et al. 2018	Retrospective cohort	253 (28)	IIIC-IV	Mayo clinic, USA	Qualitative assessment score 4 or 5, >7 mm. and ≥10 mm. in short axis diameter on CT scan	To investigate the prognostic significance of enlarged CPLN detected by preoperative CT scan.	Enlarged CPLN was associated with decreased OS (median OS 38.4 vs 69.6 months P=0.08). No association between enlarged CPLN and OS among patients with residual disease (RD) was observed (median OS 37.5 vs 28.5 months, p=0.49). There was no difference in OS between patients with NGR VS RD when enlarged CPLN were present (median OS 38.4 vs 37.5 months, p=0.99)
34	Nuruzzaman Md. S. H et al. 2018	Case report	1 (1)	IVB	Singapore general hospital	-	To report case of complete cytoreduction including CPLN excision.	Transdiaphragmatic excision of CPLN is feasible without major complication. No correlation with OS or PFS yet.
28	Salehi S. et al. 2018	Retrospective cohort	180 (24)	IIIC-IV	Skane University Hospital, Lund, Sweden	>8mm. in short axis diameter or heterogenous enhancement or absence of fatty hilum or a lobulated margin	To evaluate the accuracy of preoperative imaging in the diagnosis of CPLN metastases and to report perioperative outcomes after resection of CPLN	20/24 had confirmed metastases upon final pathology. CPLN resection is highly feasible without considerable added morbidity.
11	Lopes A. et al. 2019	Retrospective cohort	456 (14)	IIIc-IV	The Instituto Brasileiro de Controle do Cancer and the Instituto do Cancer do Estado de Sao Paulo, Brazil	> 8mm. in short axis on CT or MRI or 18-fluoroine fluorodeoxyglucose uptake on PET-CT	To evaluate the clinical outcomes of patients who underwent CPLN resection	21/24 patients had CPLN positive. The median PFS and OS was 12 (95%CI 7.2-16.7) months and 30 (95%CL 22.5 to 37.4) months, respectively.
29	Prader S. et al. 2019	Retrospective cohort	350 (217)	IIIB-IV	Kliniken Essen-Mitte, Germany	≥5mm in short axis diameter on CT scans	To evaluate the pattern of CPLN metastases, their prognostic impact and the potential role of CPLN resection.	In patient with complete resection, negative CPLN had a 5-year OS 69% vs positive CPLN had 5-year OS 30%. Patients with negative CPLN had a 5-year PFS of 41% positive CPLN vs 13%. 52/217 who were resected CPLN match pair case control analysis did not show any significant increase of OS after CPLN resection.
35	Pinelli C. et al. 2019	Case series	7 (7)	IV	Churchill Hospital Oxford, UK	≥7 mm in short axis diameter on CT scans	To investigate the accuracy and feasibility of CPLN assessment and removal during IDS after NACT	The result confirms that CPLN resection can be performed safely in the context of IDS when is essential to achieve an optimal cytoreduction.
31	Luger K. A. et al. 2020	Retrospective cohort	178 (89)	III-IV	Innsbruck medical University, Austria	≥5 mm. in short axis diameter on CT scan	To evaluate the clinical impact of preoperative radiologic assessment of CPLN status.	CPLN≥5mm revealed independent prognostic relevance for PFS HR2.14 (95%CI1.33-3.42) and OS (HR2.18 95%CI1.16-4.08). Patients with enlarged CPLN nonetheless benefit from complete intra-abdominal tumor debulking in terms of an improvement in PFS HR 0.60 (95%CI 0.38-0.94) and OS HR 0.59 95%CI 0.35-0.82).
32	Oommen I. et al.2020	Retrospective cohort	101 (42)	I-IV	Christian Medical College, India	≥7 mm in short axis diameter on CT scans	To assess the relevance of enlarged Cardiophrenic lymph	Enlarged CPLN was significantly associated with higher radiological PCI (p=0.002), large volume upper abdominal disease (p=0.001), enlarged lesser omental, periportal and suprarenal para-

nodes seen on staging CT.	aortic lymph nodes ($p \le 0.05$), unfavorable sites of disease involvement ($p<0.001$), and extraperitoneal metastases ($p=0.004$). CT detected enlarge CPLN did not adversely affect OS, HR 1.5, $p=0.272$ but adversely affected PFS, HR2.38, $p=0.008$

Table S4. The association between radiologically suspicious CPLN and histologically confirmed CPLN. CPLN; Cardiophrenic lymph nodes, *Patients underwent neoadjuvant chemotherapy 3-6 cycles before debulking surgery.

Study	Number of patients with CPLN resection	Definition of CPLN adenopathy on short axis CT scan (mm)	Mean size of CPLN (mm)	SD	Histological confirmation (%)
Kim H. T. et al. 2016	31	>7	7	2.26	19 (61.3)
Cowan R. A. et al. 2017	54	>5	15	0.5	51(94.4)
Lee O. I. et al. 2018	25	FDG uptake	8.05	2.6	21(84)
Salehi S. et al. 2018	24	>8	8.8	2.7	20(83.3)
Prader S. et al. 2019	52	≥5	8.5	2.9	44(84.6)
Pinelli C. et al. 2019*	7	>7 and intraoperative bulky nodes	-	-	4(57.14)

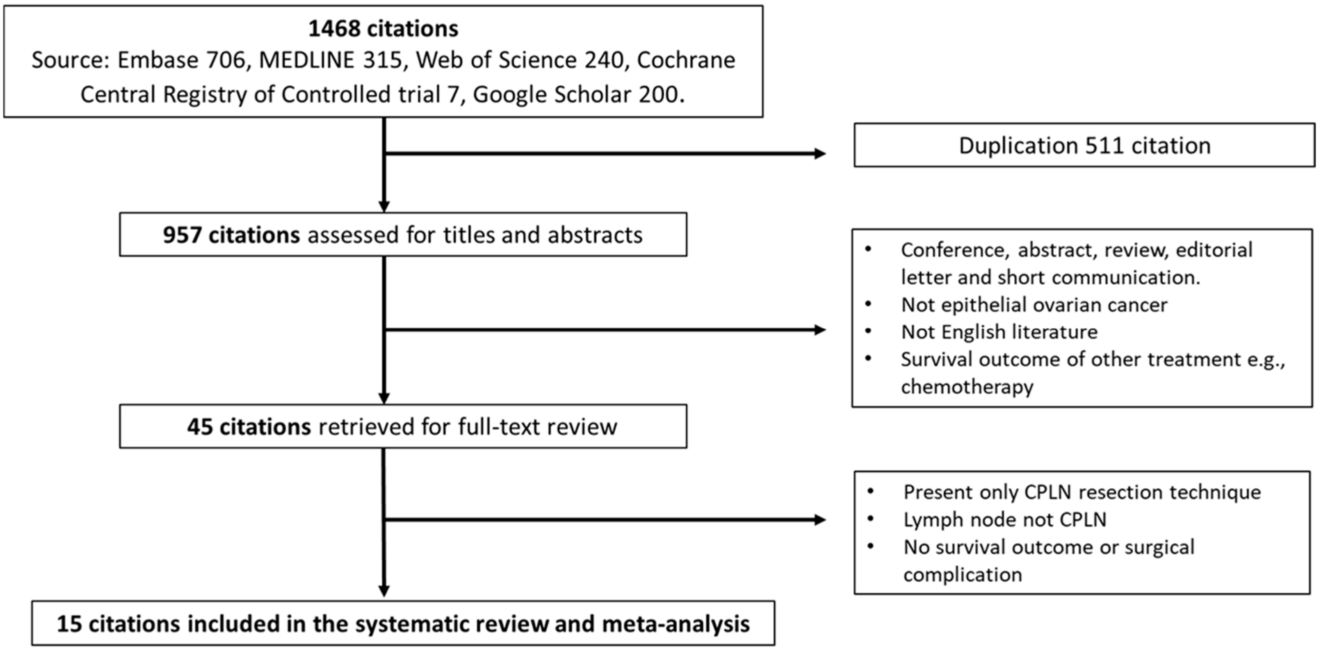

Figure S1. Flow diagram of study selection.

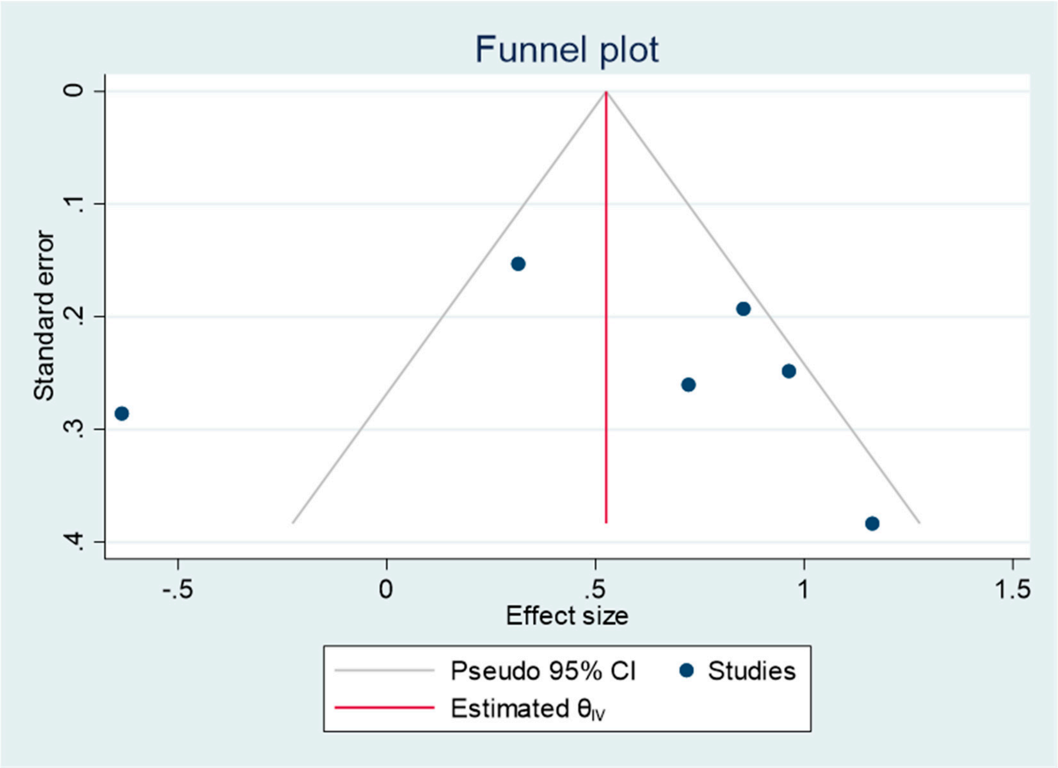

Figure S2. Funnel plot, using data from 6 studies of 5-year mortality in advanced stage EOC with CPLN adenopathy.

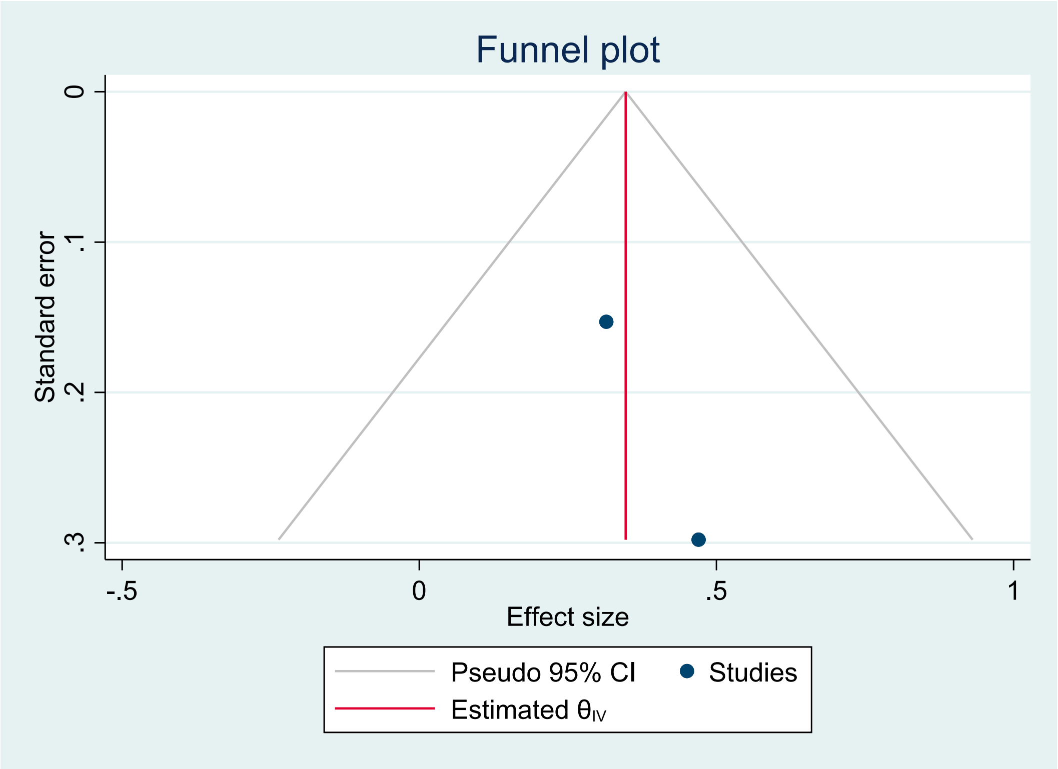

Figure S3. Funnel plot, using data from 2 studies of 5-year mortality in advanced stage EOC with CPLN adenopathy after optimal abdominal cytoreductive surgery.

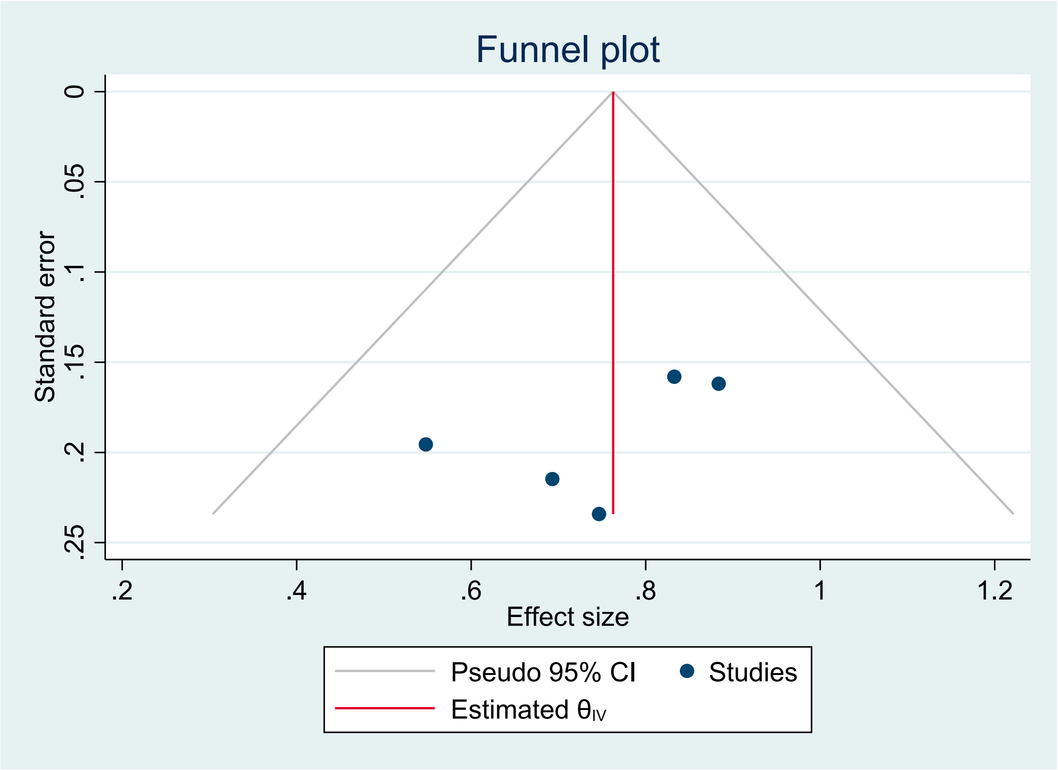

Figure S4. Funnel plot, using data from 5 studies of 5-year disease free survival in advanced stage EOC with CPLN adenopathy.
